# ATF3 Prevents Stress-Induced Hematopoietic Stem Cell Exhaustion

**DOI:** 10.3389/fcell.2020.585771

**Published:** 2020-10-27

**Authors:** Yufeng Liu, Yingying Chen, Xiaohui Deng, Jie Zhou

**Affiliations:** ^1^Joint Program in Immunology, Department of Internal Medicine, Affiliated Guangzhou Women and Children’s Medical Center, Zhongshan School of Medicine, Sun Yat-sen University, Guangzhou, China; ^2^Institute of Human Virology, Zhongshan School of Medicine, Sun Yat-sen University, Guangzhou, China; ^3^Department of Immunology, Key Laboratory of Immune Microenvironment and Disease of Ministry of Education, School of Basic Medical Sciences, Tianjin Medical University, Tianjin, China

**Keywords:** ATF3, hematopoietic stem cells, self-renewal, stress, bone marrow transplantation

## Abstract

Protection of hematopoietic stem cells (HSCs) from exhaustion and effective regeneration of the HSC pool after bone marrow transplantation or irradiation therapy is an urgent clinical need. Here, we investigated the role of activating transcription factor 3 (ATF3) in steady-state and stress hematopoiesis using conditional knockout mice (*Atf3*^*fl/fl*^Vav1*Cre* mice). Deficiency of ATF3 in the hematopoietic system displayed no noticeable effects on hematopoiesis under steady-state conditions. Expression of ATF3 was significantly down-regulated in long-term HSCs (LT-HSCs) after exposure to stresses such as 5-fluorouracil challenge or irradiation. *Atf3*^*fl/fl*^Vav1*Cre* mice displayed enhanced proliferation and expansion of LT-HSCs upon short-term chemotherapy or irradiation compared with those in *Atf3*^*fl/fl*^ littermate controls; however, the long-term reconstitution capability of LT-HSCs from *Atf3*^*fl/fl*^Vav1*Cre* mice was dramatically impaired after a series of bone marrow transplantations. These observations suggest that ATF3 plays an important role in preventing stress-induced exhaustion of HSCs.

## Introduction

Hematopoietic stem cells (HSCs) possess the capability to self-renew and produce mature cells to replenish the blood system for lifelong hematopoiesis (Arai et al., 2004; Yoshihara et al., 2007, Orkin and Zon, 2008). Under steady state conditions, HSCs predominantly exist in a quiescent state (Wilson et al., 2008). Under hematopoietic stress conditions such as infections, chemotherapy, or transplantation, HSCs exit quiescence, rapidly proliferate, and differentiate into mature cells to replenish blood cell numbers through hematopoiesis (Essers et al., 2009; Baldridge et al., 2010). A defect in the capability of HSCs to exit quiescence results in insufficient production of blood cells, whereas failure to re-enter quiescence after stress causes functional exhaustion of HSCs (Hou et al., 2015). Therefore, the homeostasis of HSCs depends on maintaining the balance between quiescence and activation. The mechanism by which HSCs orchestrate this balance remains unclear in the field of stem cell biology. Some transcription factors that participate in metabolism, cell cycle, and epigenetic modifications have been demonstrated to regulate HSC function (Cheung and Rando, 2013). In addition to intrinsic cell mechanisms, HSCs are tightly regulated by extrinsic mechanisms, such as signaling provided by mesenchymal stem cells, non-myelinating Schwann cells, and megakaryocytes residing in the bone marrow microenvironment (Mendelson and Frenette, 2014; Zhao et al., 2014).

Activating transcription factor 3 (ATF3) is a basic-region leucine zipper transcription factor belonging to the cyclic AMP response element binding family. ATF3 plays important role in cellular responses to stresses by dictating the expression of genes involved in the cell cycle, DNA repair, or cell survival (Thompson et al., 2009). Its importance in defending against invading pathogens, and suppressing inflammatory responses has been well-documented (Hai et al., 2010). ATF3 has been identified as a negative regulator of Toll-like receptor signaling in macrophages (Hoetzenecker et al., 2011). Mice with ATF3 deficiency displayed enhanced susceptibility to endotoxin shock induced sepsis (Lai et al., 2013). ATF3-deficient mice showed aggravated allergic airway inflammation due to higher Th2 responses in the lung (Gilchrist et al., 2008). In the intestine, induction of ATF3 can prevent the development of colitis by facilitating the development of follicular helper T cells and microbiota homeostasis (Cao et al., 2019, 2020). These studies indicate that the induction of ATF3 through environmental stimuli represents a protective mechanism in the host to avoid inflammation. However, the role of ATF3 in stress hematopoiesis remains largely unknown.

Here, we showed that ATF3 was dispensable for normal hematopoiesis, and that stress induced dramatic down-regulation of ATF3 in HSCs. ATF3 deficiency in the hematopoietic system enhanced the proliferation of LT-HSCs after short-term challenges with 5-fluorouracil (FU) or irradiation, which led to functional exhaustion of HSCs after long-term bone marrow transplantation. These observations indicate that ATF3 plays a protective role in stress hematopoiesis to maintain HSC self-renewal.

## Materials and Methods

### Animals

*Atf3*^*flox/flox*^ mice were generated at Nanjing Biomedical Research Institute of Nanjing University (Jiangsu, China) as described previously (Cao et al., 2019), and then crossed with *Vav1-Cre* transgenic mice (Jackson Laboratory, Bar Harbor, ME, United States) to produce conditional knockout mice (*Atf3*^*fl/fl*^Vav1*Cre*). Their littermates (*Atf3*^*fl/fl*^) were used as controls. C57BL/6SJL (CD45.1^+^) mice were kindly provided by Dr. Haikun Wang (Chinese Academy of Sciences, Shanghai, China). The recipient mice used in bone marrow transplantation assays were (CD45.1/45.2^+^) heterozygotes, which were obtained by crossbreeding of wild-type (WT) (C57BL/6 background, CD45.2^+^) crossed with C57BL/6SJL (CD45.1^+^) mice. All mice were 8–10 weeks old and maintained in a specific pathogen-free animal facility. All animal experiments in this research were conducted according to protocols approved by The Animal Care and Ethics Committee of Tianjin Medical University.

### Flow Cytometry

Bone marrow (BM) cells isolated from the femur and tibia were prepared as described previously (Lei et al., 2018). All collected cells were treated with ACK (red blood cell lysis) buffer before analysis. To analyze hematopoietic cells, monoclonal antibodies from BD Biosciences (San Jose, CA, United States), eBioscience (San Diego, CA, United States), and BioLegend (San Diego, CA, United States) recognizing the following surface markers were used: Sca-1 (D7), c-Kit (2B8), CD150 (mShad150), CD48 (HM48-1), CD127 (A7R34), CD16/32 (93), Gr-1 (RB6-8C5), Mac-1 (M1/70), B220 (RA3-6B2), CD3e (145-2C11), CD45.1 (A20), CD45.2 (104), Ki67 (7B11), and streptavidin. The mouse lineage cocktail contained anti-CD3, Mac-1, Gr-1, B220, Ter-119 (TER-119), and NK1.1 (PK136) antibodies (eBioscience). Cell analysis was performed using a FACS LSR Fortessa (BD Biosciences) and cell sorting was performed using a FACS AriaIII (BD Biosciences). Data were analyzed using FlowJo10.0 software (TreeStar, Ashland, OR, United States).

### Transplantation Assays

Recipient mice were treated with antibiotic-containing water (0.5 mg/mL amoxicillin) 1 week before and 1 week after transplantation. For competitive transplantation assays, the indicated numbers of LT-HSCs from *Atf3*^*fl/fl*^ and *Atf3*^*fl/fl*^Vav1*Cre* mice (CD45.2^+^) were mixed with 1 × 10^6^ BM cells from WT mice (CD45.1^+^) and transplanted into lethally irradiated (10 Gy) recipient mice (CD45.1/2^+^) by tail vein injection. A total of 1 × 10^6^ BM cells obtained from previous recipient mice were transplanted into lethally irradiated (10 Gy) secondary or tertiary WT (CD45.1/2^+^) recipients. Donor reconstitution was evaluated every 4 weeks.

### 5-FU Treatment and Sublethal Irradiation

*Atf3*^*fl/fl*^ and *Atf3*^*fl/fl*^Vav1*Cre* mice were intraperitoneally injected (*i.p.*) with a single dose of 5-FU (150 mg/kg; Sigma-Aldrich, St. Louis, MO, United States). To administer a sublethal dose of radiation, the mice were irradiated with 5 Gy total body radiation. Blood cells were counted, and hematopoietic stem and progenitor cells (HSPCs) were analyzed by flow cytometry.

### LPS Treatment

Mice were injected *i.p.* with phosphate-buffered saline or 0.25 mg/kg lipopolysaccharide (LPS; *Escherichia coli* 0111:B4; Sigma Aldrich), and the dynamic expression of ATF3 in LSKs or LT-HSCs was determined by flow cytometry or quantitative reverse-transcription polymerase chain reaction (qRT-PCR).

### Cell Cycle Analysis and BrdU Incorporation Assay

The cell cycle was analyzed by Ki67 (1:100 in BD Perm/Wash buffer for 30 min) and DAPI (2 mg/mL for 10 min) staining using Cytofix/Cytoperm Fixation/Permeabilization Kit (BD Biosciences). For the BrdU incorporation assay (Hu et al., 2018), the mice were injected *i.p.* with 100 mg/kg BrdU (BD Biosciences) at 12 h before harvesting the BM. BrdU Flow kit (BD Biosciences) was used according to the manufacturer’s protocol.

### Homing Assay

Freshly isolated BM cells from *Atf3*^*fl/fl*^ and *Atf3*^*fl/fl*^Vav1*Cre* mice were labeled with 5- and 6-carboxyfluorescein succinimidyl ester (CFSE; eBioscience). Next, 1 × 10^7^ BM cells were transplanted into lethally irradiated (10 Gy) recipient mice. At 16 h later, CFSE^+^ LSKs in the BM of recipient mice were analyzed by flow cytometry (Cancelas et al., 2005).

### Complete Blood Cell Count

A hematology system (Hemavet 950FS; Drew Scientific, Miami Lakes, FL, United States) was used to analyze the complete blood cell count from the total blood collected from the mice.

### qRT-PCR

Total RNA was isolated using TRIzol (Invitrogen, Carlsbad, CA, United States) and reverse-transcribed with a synthesis kit (Takara, Shiga, Japan). qRT-PCR was performed with SYBR Green (TaKaRa). The primer sequences are listed in Supplementary Table 1.

### Colony-Forming Unit (CFU) Assay

A total of 1000 LSKs was sorted and plated in 1 mL of mouse MethoCult 3434 (Stem Cell Technologies, Vancouver, Canada) and then cultured at 37°C. Colonies were counted with an inverted microscope on day 14.

### BM Culture

BM cultures were performed as described previously (Singh et al., 2013). In brief, 2 × 10^5^ cells of total nucleated BM cells were cultured in Iscove’s Modified Dulbecco’s Medium (IMDM) medium with 10% serum in presence of the indicated growth factors, 100 ng/ml murine SCF, 50 ng/ml murine IL6, 100 ng/ml murine TPO, and 100 ng/ml murine FLT3L (all cytokines form PeproTech, Rocky Hill, United States) for 10 days.

### Statistical Analysis

Data are presented as the means ± standard deviation from two independent experiments. Differences between two groups were determined by unpaired Student’s *t* tests, and multiple groups were assessed by one-way analysis of variance with Tukey–Kramer *post hoc* analysis. Mann–Whitney U tests were used when the criteria for normal distribution were not satisfied. The survival curve was assessed by a log-rank (Mantel–Cox) test. All statistical analyses were performed with GraphPad Prism 8.0 software (GraphPad Software, Inc., CA, United States). Statistical significance is indicated by ^∗^*P* < 0.05; ^∗∗^*P* < 0.01; and ^∗∗∗^*P* < 0.001.

## Results

### ATF3 Is Dispensable for Steady-State Hematopoiesis

**FIGURE 1:**
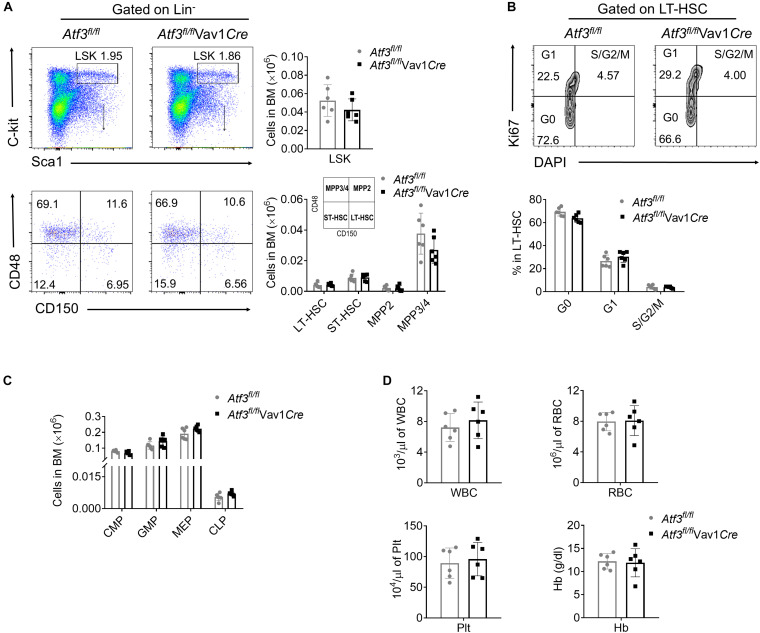
ATF3 is dispensable for steady-state hematopoiesis **(A)** Representative flow cytometric plots (left) and absolute numbers (right) of LSKs (lineage^–^Sca-1^+^C-Kit^+^), LT-HSCs (CD150^+^CD48^–^LSKs), ST-HSCs (CD150^–^CD48^–^LSKs), MPP2 (CD150^+^CD48^+^LSKs), and MPP3/4 (CD150^–^CD48^+^LSKs) in the BM cells of *Atf3*^*fl/fl*^ and *Atf3*^*fl/fl*^ Vav1*Cre* mice (*n* = 6). **(B)** Representative flow cytometric plots and percentages of cell cycle distribution of LT-HSCs (CD150^+^CD48^–^LSK) in the BM cells of *Atf3*^*fl/fl*^ and *Atf3*^*fl/fl*^Vav1*Cre* mice (*n* = 6). **(C)** Absolute numbers of GMPs (CD34^+^CD16/32^+^Lineage^–^C-Kit^+^Sca-1^–^), CMPs (CD34^+^CD16/32^–^Lineage^–^C-Kit^+^Sca-1^–^), MEPs (CD34^–^CD16/32^–^Lineage^–^C-Kit^+^Sca-1^–^), and CLPs (CD127^+^Flk2^+^Lineage^–^Sca-1^*low*^C-Kit^*low*^) in the BM cells of *Atf3*^*fl/fl*^ and *Atf3*^*fl/fl*^Vav1*Cre* mice (*n* = 6). **(D)** Complete blood cell counts including WBC, RBC, Plt, and Hb from *Atf3*^*fl/fl*^ and *Atf3*^*fl/fl*^Vav1*Cre* mice (*n* = 6). Data are representative of two independent experiments. Error bars show mean ± SD.

To explore the role of ATF3 in hematopoiesis, ATF3 in the hematopoietic system was specifically deleted by cross-breeding *Atf3*^*flox/flox*^ mice with *Vav1-Cre* mice to generate conditional knock-out mice, named as *Atf3*^*fl/fl*^Vav1*Cre* (Supplementary Figure 1A). The frequencies of HSPCs in *Atf3*^*fl/fl*^Vav1*Cre* mice were determined by flow cytometry. The frequencies of distinct HSPCs, including LSKs (Lin^–^C-kit^+^Sca-1^+^), long-term HSCs (LT-HSCs), short-term HSCs (ST-HSCs), and MPPs, were comparable between *Atf3*^*fl/fl*^Vav1*Cre* and *Atf3*^*fl/fl*^ littermate controls (Figure 1A and Supplementary Figure 1B). Cell cycling analysis revealed no changes in the proliferation of LT-HSCs under steady state conditions (Figure 1B). The frequencies of lineage-determined progenitors, including common myeloid progenitors (CMPs), granulocyte monocyte progenitors (GMPs), megakaryocyte erythroid progenitors (MEPs), and common lymphoid progenitors (CLPs), were also comparable between *Atf3*^*fl/fl*^Vav1*Cre* and *Atf3*^*fl/fl*^ mice (Figure 1C). The complete blood cell counts, including white blood cell (WBC), red blood cell (RBC), platelet (Plt), and hemoglobin (Hb), failed to differ between *Atf3*^*fl/fl*^Vav1*Cre* and *Atf3*^*fl/fl*^ littermates (Figure 1D). These results indicate that ATF3 is dispensable for maintaining steady-state hematopoiesis.

### ATF3 Is Down-Regulated in HSCs Upon Stress

**FIGURE 2:**
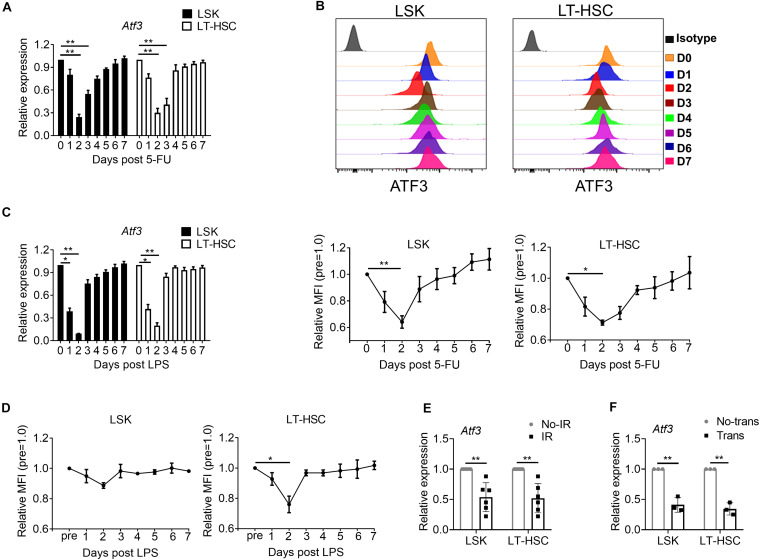
ATF3 is down-regulated in HSCs upon hematological stress. **(A)** qRT-PCR analysis of *Atf3* mRNA expression in LSKs and LT-HSCs from WT BM cells after 5-FU treatment (150 mg/kg) (*n* = 4–6). **(B)** Representative flow cytometry histogram (upper) and mean fluorescence intensity (lower) of ATF3 expression in LSKs (left) and LT-HSCs (right) from WT BM cells at the indicated time points after 5-FU (150 mg/kg) treatment (*n* = 3–5). **(C)** qRT-PCR analysis of *Atf3* mRNA expression in LSKs and LT-HSCs from WT BM cells after LPS treatment (0.25 mg/kg) (*n* = 4–6). **(D)** Mean fluorescence intensity (MFI) of ATF3 expression in LSKs (left) and LT-HSCs (right) from WT BM cells at the indicated time points after LPS treatment (0.25 mg/kg) (*n* = 3–5). **(E,F)** *Atf3* mRNA level determined by qRT-PCR in WT LSKs and LT-HSCs isolated 24 h after irradiation (IR, E, *n* = 6) and transplantation (Trans, F, *n* = 3). For irradiation, mice were irradiated at a sublethal dose of 5 Gy, and control mice without irradiation. For transplantation, 1 × 10^3^ LT-HSCs from WT mice (CD45.2^+^) were mixed with WT BM cells (1 × 10^6^, CD45.1^+^), followed by transplantation into lethally irradiated congenic recipients (CD45.1/2^+^), and control mice without donor cell transplantation. Data are representative of two independent experiments. Error bars show the mean ± SD. *P* values was determined using two-sided Student’s *t* tests (E, F) and one-way analysis of variance followed by Tukey-Kramer multiple-comparisons test (A–D). *P < 0.05; **P < 0.01.

To determine whether ATF3 plays a role under stress-induced hematopoiesis, we evaluated its expression in HSPCs upon hematopoietic stress. WT mice were injected with the cytotoxic reagent 5-FU at a single dose (150 mg/kg weight) for 7 days, after which the dynamic expression of ATF3 was evaluated. ATF3 was substantially down-regulated in both LSKs and LT-HSCs at day 2 after 5-FU injection and gradually rescued thereafter at the mRNA (Figure 2A) and protein levels (Figure 2B). The dynamic expression pattern of ATF3 was not observed in lineage-committed myeloid progenitors (MPs) (Supplementary Figure 2). Administration with lipopolysaccharide, another cytotoxic reagent, induced similar effects (Figures 2C,D). For further confirmation, WT mice were irradiated at a sublethal irradiation of 5 Gy, and LSKs or LT-HSCs were isolated after 24 h. It was shown that mRNA expression of *Atf3* was lower in irradiated mice when compared with those from non-irradiated controls (Figure 2E). Furthermore, 1 × 10^3^ LT-HSCs from WT mice (CD45.2^+^) were mixed with WT BM cells (1 × 10^6^, CD45.1^+^), followed by transplantation into lethally irradiated congenic recipients (CD45.1/2^+^). LSKs or LT-HSCs were isolated after 24 h. It was found that *Atf3* was substantially down-regulated in cells from transplanted recipients (Figure 2F). These observations suggest that stress-induced proliferation of HSCs is accompanied by down-regulation of ATF3 in HSCs.

### Loss of ATF3 Facilitates HSC Proliferation and Expansion in Response to 5-FU Treatment

**FIGURE 3:**
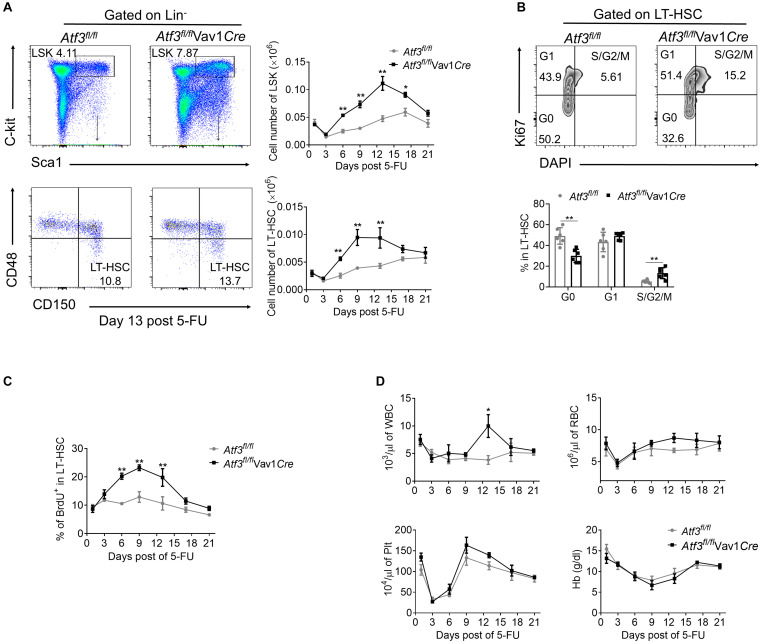
Loss of ATF3 leads to increased HSC proliferation and expansion in response to 5-FU treatment. **(A)** Representative FACS plots of LSKs and LT-HSCs from *Atf3*^*fl/fl*^ and *Atf3*^*fl/fl*^Vav1*Cre* mice at 13 days after 5-FU treatment (left). The absolute numbers of LSKs and LT-HSCs at the indicated time points after 5-FU injection were determined by flow cytometry (right, *n* = 6). **(B)** Representative FACS analysis cell cycle of LT-HSC (CD150^+^CD48^–^LSK) from *Atf3*^*fl/fl*^ and *Atf3*^*fl/fl*^Vav1*Cre* mice at day 9 after 5-FU treatment (*n* = 6). **(C)** The percentages of BrdU^+^ cells in LT-HSC (CD150^+^CD48^–^LSK) at the indicated time points after 5-FU injection were determined by flow cytometry (*n* = 6–7). **(D)** Complete blood cell counts were analyzed by a Hemavet 950FS hematology system after 5-FU injection (*n* = 5–10). Data are representative of two independent experiments. Error bars show mean ± SD. *P* values was determined using two-sided Student’s *t* tests. *P < 0.05; **P < 0.01.

We next examined whether a functional link exists between decreased expression of ATF3 and HSC functionality upon hematopoietic stress. It is known that 5-FU injury causes rapid loss of cycling hematopoietic cells at initiation, followed by recovery of hematopoietic homeostasis (Xu et al., 2017). As expected, depletion of LSKs and LT-HSCs before day 3 post 5-FU challenge was observed, followed by recovery from day 6 and peaked around day 12 in both *Atf3*^*fl/fl*^Vav1*Cre* and *Atf3*^*fl/fl*^ mice (Figure 3A and gating strategy shown in Supplementary Figure 3A). However, the absolute numbers of LSKs and LT-HSCs from *Atf3*^*fl/fl*^Vav1*Cre* mice were significantly higher than those from *Atf3*^*fl/fl*^ littermates during 5-FU challenge (Figure 3A). According to Ki67 and DAPI staining, LT-HSCs from *Atf3*^*fl/fl*^Vav1*Cre* mice displayed much lower levels of quiescence (G0 phase) than *Atf3*^*fl/fl*^ controls; in contrast, HSCs in S/G2/M phase (proliferation) were significantly higher in *Atf3*^*fl/fl*^Vav1*Cre* mice (Figure 3B). The BrdU incorporation assay confirmed the higher proliferative capability of LT-HSCs in *Atf3*^*fl/fl*^Vav1*Cre* mice (Figure 3C). This appreciable proliferative potential of LT-HSCs led to increased levels of WBC in *Atf3*^*fl/fl*^Vav1*Cre* mice during recovery after 5-FU treatment (Figure 3D). Alternatively, we used sublethal irradiation as a non-pharmacological toxic insult. In agreement with the observations from 5-FU treatment, *Atf3*^*fl/fl*^Vav1*Cre* mice displayed higher frequencies of LT-HSCs with enhanced proliferation (Supplementary Figures 3B–D), which led to faster recovery of hematopoiesis upon irradiation (Supplementary Figure 3E). Furthermore, BM cells from *Atf3*^*fl/fl*^ or *Atf3*^*fl/fl*^Vav1*Cre* mice were cultured in medium containing stem cell factor (SCF), Flk-2/Flt3 ligand (FLT3L), thrombopoietin (TPO), and IL-6. These four cytokine combinations were reported to promote the proliferation of multipotent hematopoietic progenitor cells *in vitro* (Zhang and Lodish, 2008). It was found that deficiency of ATF3 resulted in significant increase in the percentages and cell proliferation of HSPCs (Supplementary Figures 3F,G). These observations indicate that ATF3 deficiency facilitates the proliferation of HSCs under short-term stress.

### Loss of ATF3 Impairs Long-Term Self-Renewal of HSCs

**FIGURE 4:**
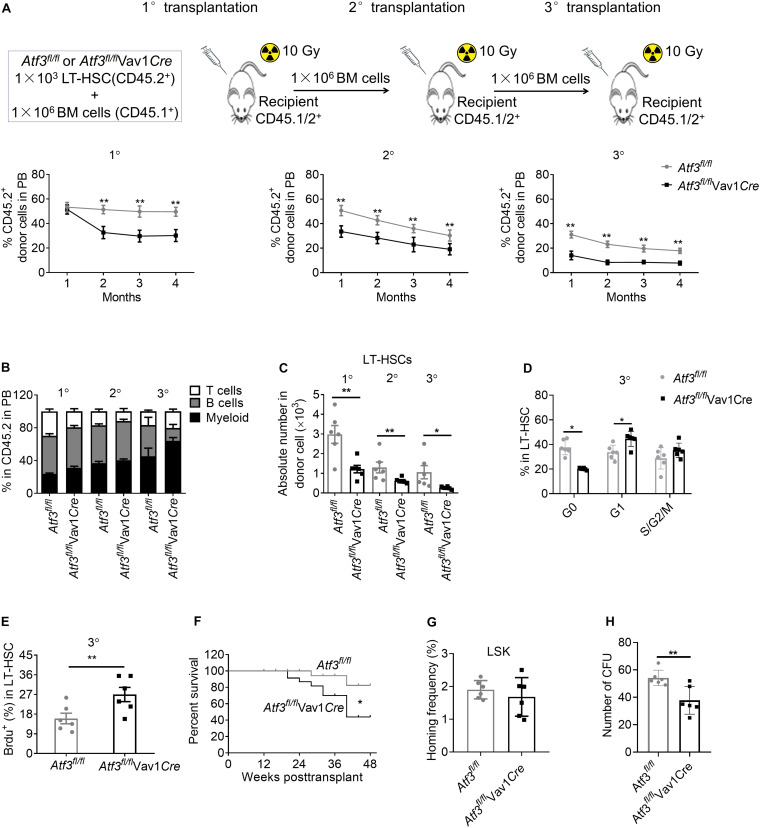
Loss of ATF3 impairs the long-term self-renewal of HSCs. **(A)** A total of 1 × 10^3^ LT-HSCs from *Atf3*^*fl/fl*^ and *Atf3*^*fl/fl*^Vav1*Cre* mice (CD45.2^+^) was mixed with competitor BM cells (CD45.1^+^) and injected into lethally irradiated recipients (CD45.1/2^+^). Four months later, the chimeric BM cells were re-transplanted into secondary or tertiary recipients (CD45.1/2^+^) (1°/2°/3° defined as primary, secondary, and tertiary transplantations, *n* = 6). Percentages of donor-derived cells in the peripheral blood (PB) were analyzed by flow cytometry at the indicated time points during serial BMT (*n* = 6). **(B)** Analysis of distinct lineages (CD3^+^ T cells, B220^+^ B cells, and Gr-1^+^ myeloid cells) in PB from recipients 4 months after transplantation (*n* = 6). **(C)** Absolute numbers of donor-derived LT-HSCs in the BM from recipients 4 months after transplantation (*n* = 6). **(D)** Percentages of donor-derived LT-HSCs in the BM cells in different cell cycle stages from recipients at 4 months after 3° transplantation (*n* = 6). **(E)** The percentages of BrdU^+^ LT-HSCs in the BM from recipients at 4 months after 3° transplantation (*n* = 6). **(F)** Survival curve of *Atf3*^*fl/fl*^ and *Atf3*^*fl/fl*^Vav1*Cre* recipient mice after transplantation (*n* = 28). **(G)** Homing analysis of CFSE^+^ LSKs from *Atf3*^*fl/fl*^ and *Atf3*^*fl/fl*^Vav1*Cre* BM cells 16 h after transplantation (*n* = 6). **(H)** At 4 months after 2°BMT, 1000 sorted LSKs from recipients were seeded in a colony-forming unit assay (*n* = 6). Data are representative of two independent experiments. Error bars show the mean ± SD. *P* values were determined using two-sided Student’s *t* tests. *P < 0.05; **P < 0.01.

Enhanced proliferation of HSCs enables fast recovery during short term stress hematopoiesis but can cause exhaustion of the HSC pool after long-term exposure (Sato et al., 2009; Walter et al., 2015). We next evaluated the self-renewal of HSCs following serial competitive BM transplantation (Figure 4A). A total of 1 × 10^3^ LT-HSCs from *Atf3*^*fl/fl*^Vav1*Cre* or *Atf3*^*fl/fl*^ animals (CD45.2^+^) were mixed with WT BM cells (1 × 10^6^, CD45.1^+^), followed by transplantation into lethally irradiated congenic recipients (CD45.1/2^+^). Secondary and tertiary transplantation was performed 16 weeks after the previous transplantation (1 × 10^6^ BM cells). Evaluation of the peripheral blood revealed a significant reduction in the chimerism of donor cells from *Atf3*^*fl/fl*^Vav1*Cre* mice when compared with those from *Atf3*^*fl/fl*^ controls during serial transplantation (Figure 4A). Analysis of distinct lineages showed that ATF3 deletion did not affect the overall proportions between myeloid and lymphoid lineages (Figure 4B). BM analysis showed that the absolute numbers of LT-HSCs derived from *Atf3*^*fl/fl*^Vav1*Cre* donor mice were markedly decreased when compared to those from *Atf3*^*fl/fl*^ donor controls, which was most pronounced during tertiary transplantation (Figure 4C). Cell cycle and BrdU staining analysis showed that LT-HSCs from *Atf3*^*fl/fl*^Vav1*Cre* mice displayed higher proliferative rate than their *Atf3*^*fl/fl*^ controls after the tertiary transplantation (Figures 4D,E). Meanwhile, Kaplan-Meier survival analysis revealed that recipients transplanted with *Atf3*^*fl/fl*^Vav1*Cre* LT-HSCs displayed significantly lower survival rate when compared with those transplantation with *Atf3*^*fl/fl*^ LT-HSCs (Figure 4F). These results indicate that *Atf3*^*fl/fl*^Vav1*Cre* HSCs tend to be exhausted after stress, and therefore cannot protect against BM transplantation. Furthermore, LSKs from *Atf3*^*fl/fl*^Vav1*Cre* displayed similar homing ability with *Atf3*^*fl/fl*^ controls when analyzed 16 h after transplantation (Figure 4G), suggesting that ATF3 did not affect migration and homing of HSCs. In addition, we performed *in vitro* CFU experiments by sorting LSKs from donors (*Atf3*^*fl/fl*^ or *Atf3*^*fl/fl*^Vav1*Cre*) after secondary BM transplantation. The *in vitro* reconstitution ability of AFT3-deficient HSCs was significantly reduced after long-term stress hematopoiesis (Figure 4H). Taken together, these observations demonstrate that loss of ATF3 impairs the long-term self-renewal of HSCs.

## Discussion

The maintenance of HSC homeostasis and function is crucial for replenishing somatic cells that are lost under physiological or stress conditions (Ito and Suda, 2014). ATF3 is a stress-responsive gene, and preliminary work suggested ATF3 as a novel regulator of HSC development (McKinney-Freeman et al., 2012). However, the physiological role of ATF3 in HSC biology remained unknown. Here, using ATF3 hematopoietic system condition-knockout mice (*Atf3*^*fl/fl*^Vav1*Cre*), we demonstrate that ATF3 is dispensable for hematopoiesis under steady-state condition but ATF3 is required for maintaining HSC quiescence and self-renewal under stress hematopoiesis.

In the present study, the absence of ATF3 failed to affect homeostatic hematopoiesis and quiescence of HSCs, but its expression was down-regulated after stress stimuli, such as 5-FU, LPS, or irradiation treatment. This down-regulation was mainly observed in primitive HSCs, indicating its potential role in stress hematopoiesis. Furthermore, deletion of ATF3 in the hematopoietic system significantly enhanced the proliferation of LT-HSCs, which resulted in faster recovery of hematopoiesis and provided benefit during short-term 5-FU challenge or irradiation (Figure 3). However, it is known that the maintenance of quiescence is critical for HSCs self-renewal function, and defective HSCs quiescence often results in HSCs exhaustion during long-time regeneration (Pietras et al., 2011). The higher proliferative advantage of LT-HSCs from *Atf3*^*fl/fl*^Vav1*Cre* mice caused impaired self-renew during long-term hematopoietic stress as represented in the competitive repopulation assays. This observations from ATF3-deficient mice were similar with the results from the absence of Sirt1-knock-out mice (Singh et al., 2013), as well as mice deficient in *Ptch2* under long-time regeneration (Klein et al., 2016). These genes collectively play important roles in the regulation of HSCs quiescence and self-renewal under stress hematopoiesis.

An important limitation of this study is the lack of mechanism research. To elucidate the molecular consequences of ATF3 loss in HSC regulation, we plan to perform genome-wide expression analysis using RNA-sequence of purified *Atf3*^*fl/fl*^ and *Atf3*^*fl/fl*^Vav1*Cre* LT-HSCs derived from short and long-time hematopoietic stress, further determining the critical role of ATF3 in the gene regulatory networks governing HSC fate during stress hematopoiesis. Meanwhile, single human cord blood unit containing limited number of HSCs has been an obstacle for clinical applications, such as HSC transplantation (Walasek et al., 2012). Our observation that loss of ATF3 resulting in decrease of HSC regeneration function prompted us to test whether overexpression ATF3 could facilitate human cord blood HSCs regeneration, thus, we plan to analyze BM cells from recipient NSG mice 16 weeks post transplantation and measure the functional HSC frequency after *in vitro* expansion by transplanting GFP^+^ cells sorted from human cord blood CD34^+^ cells infected with control and overexpression ATF3 lentivirus.

In summary, this study revealed ATF3 as an important regulator of HSCs self-renewal under stress, which may have therapeutic value in the hematopoietic repopulation after chemotherapy or bone marrow transplantation.

## Data Availability Statement

The datasets used and/or analyzed during the current study are available from the corresponding author on reasonable request.

## Ethics Statement

All animal experiments in this research were conducted according to protocols permitted by The Animal Care and Ethics Committee of Tianjin Medical University.

## Author Contributions

YL and YC designed and performed most experiments and analyzed the data. XD participated in the experiments. JZ conceptualized, supervised, interpreted the experiments, and wrote the manuscript. All authors contributed to the article and approved the submitted version.

## Conflict of Interest

The authors declare that the research was conducted in the absence of any commercial or financial relationships that could be construed as a potential conflict of interest.
