# Supplementary material for: ATF3 Prevents Stress-Induced Hematopoietic Stem Cell Exhaustion
Source: Front Cell Dev Biol. 2020 Oct 27;8:585771. doi: 10.3389/fcell.2020.585771 (PMC7652754; doi:10.3389/fcell.2020.585771)
Supplement: Supplementary file 1 [file Presentation_1.PPTX]

## Slide 1
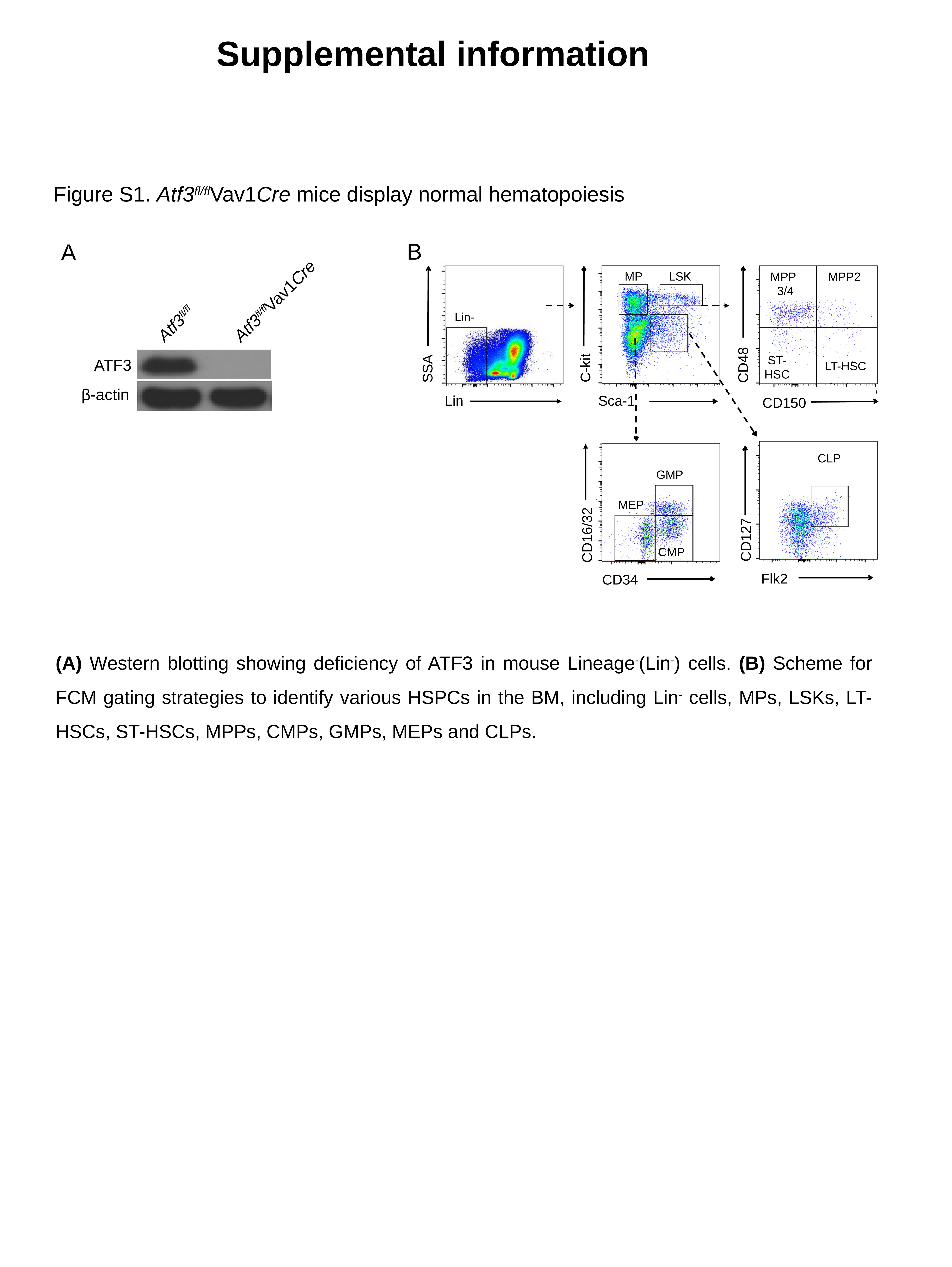

Supplemental information
Figure S1. Atf3fl/flVav1Cre mice display normal hematopoiesis
B
MP
LSK
MPP
 3/4
MPP2
Lin-
CD48
 ST-
HSC
C-kit
LT-HSC
SSA
Lin
Sca-1
CD150
CLP
GMP
MEP
CD16/32
CD127
CMP
Flk2
CD34
A
Atf3fl/flVav1Cre
Atf3fl/fl
ATF3
β-actin
(A) Western blotting showing deficiency of ATF3 in mouse Lineage-(Lin-) cells. (B) Scheme for FCM gating strategies to identify various HSPCs in the BM, including Lin- cells, MPs, LSKs, LT-HSCs, ST-HSCs, MPPs, CMPs, GMPs, MEPs and CLPs.

## Slide 2
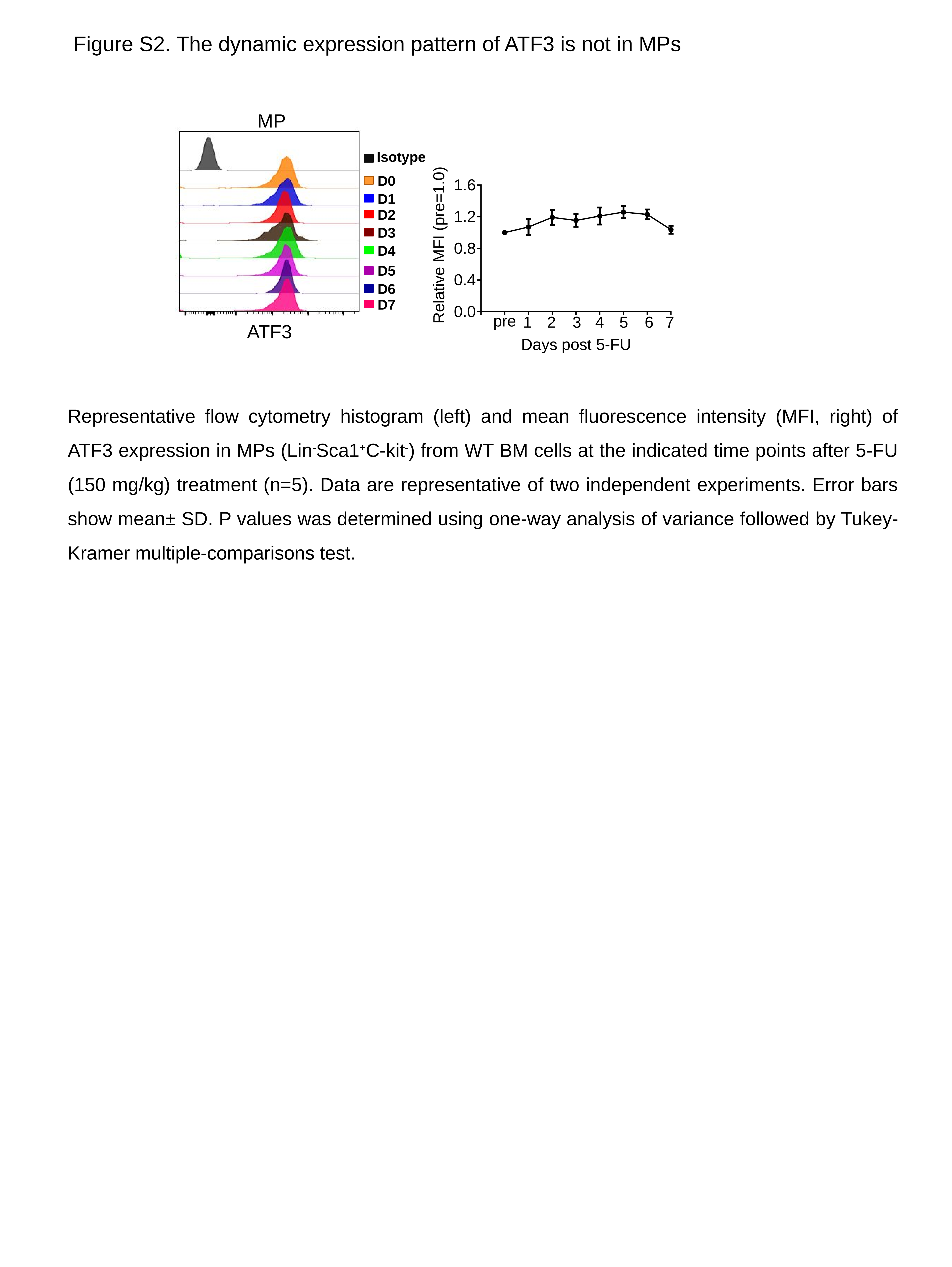

Figure S2. The dynamic expression pattern of ATF3 is not in MPs
MP
Isotype
D0
D1
D2
D3
D4
D5
D6
D7
ATF3
Representative flow cytometry histogram (left) and mean fluorescence intensity (MFI, right) of ATF3 expression in MPs (Lin-Sca1+C-kit-) from WT BM cells at the indicated time points after 5-FU (150 mg/kg) treatment (n=5). Data are representative of two independent experiments. Error bars show mean± SD. P values was determined using one-way analysis of variance followed by Tukey-Kramer multiple-comparisons test.

## Slide 3
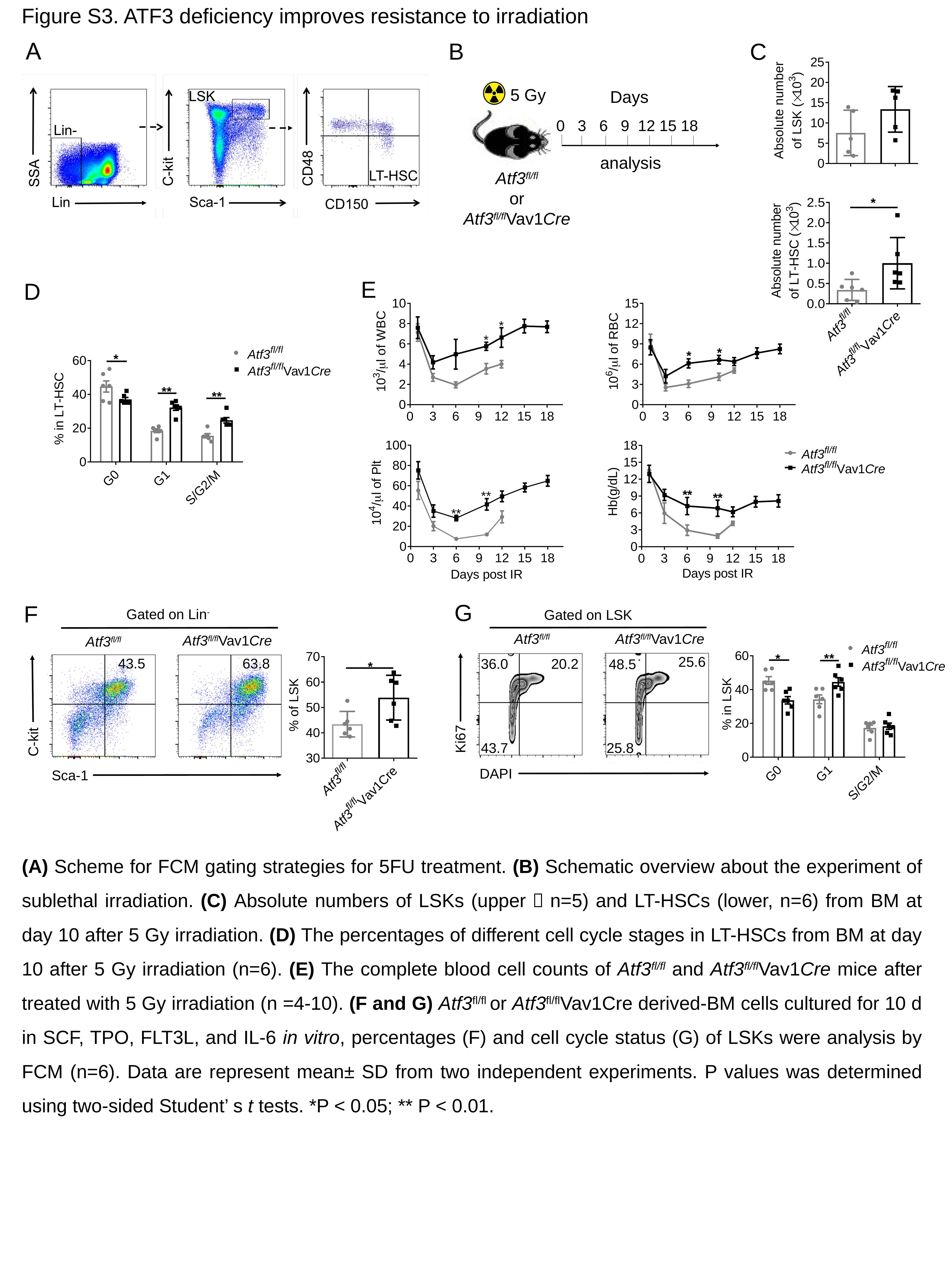

Figure S3. ATF3 deficiency improves resistance to irradiation
B
C
5 Gy
Days
0 3 6 9 12 15 18
analysis
Atf3fl/fl
or Atf3fl/flVav1Cre
E
D
G
Gated on LSK
Atf3fl/fl
Atf3fl/flVav1Cre
25.6
Ki67
20.2
36.0
48.5
43.7
25.8
DAPI
F
Gated on Lin-
Atf3fl/flVav1Cre
Atf3fl/fl
43.5
63.8
C-kit
Sca-1
(A) Scheme for FCM gating strategies for 5FU treatment. (B) Schematic overview about the experiment of sublethal irradiation. (C) Absolute numbers of LSKs (upper，n=5) and LT-HSCs (lower, n=6) from BM at day 10 after 5 Gy irradiation. (D) The percentages of different cell cycle stages in LT-HSCs from BM at day 10 after 5 Gy irradiation (n=6). (E) The complete blood cell counts of Atf3fl/fl and Atf3fl/flVav1Cre mice after treated with 5 Gy irradiation (n =4-10). (F and G) Atf3fl/fl or Atf3fl/flVav1Cre derived-BM cells cultured for 10 d in SCF, TPO, FLT3L, and IL-6 in vitro, percentages (F) and cell cycle status (G) of LSKs were analysis by FCM (n=6). Data are represent mean± SD from two independent experiments. P values was determined using two-sided Student’ s t tests. *P < 0.05; ** P < 0.01.

## Slide 4
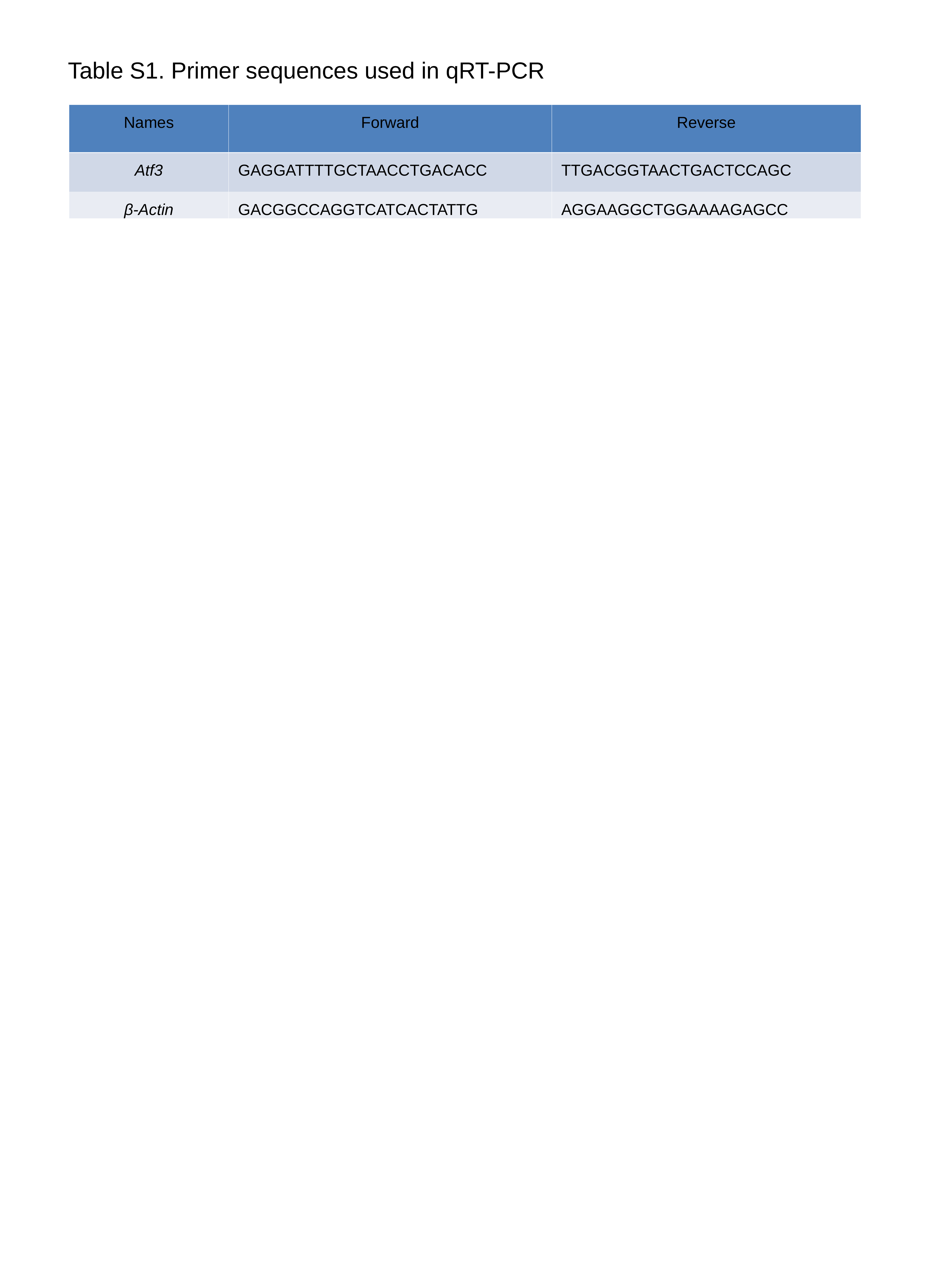

Table S1. Primer sequences used in qRT-PCR
| Names | Forward | Reverse |
| --- | --- | --- |
| Atf3 | GAGGATTTTGCTAACCTGACACC | TTGACGGTAACTGACTCCAGC |
| β-Actin | GACGGCCAGGTCATCACTATTG | AGGAAGGCTGGAAAAGAGCC |
